# The chromosome-level genome of dragon fruit reveals whole-genome duplication and chromosomal co-localization of betacyanin biosynthetic genes

**DOI:** 10.1038/s41438-021-00501-6

**Published:** 2021-03-10

**Authors:** Jinfang Zheng, Lyndel W. Meinhardt, Ricardo Goenaga, Dapeng Zhang, Yanbin Yin

**Affiliations:** 1 Nebraska Food for Health Center, Department of Food Science and Technology, University of Nebraska, Lincoln, NE 68588 USA; 2 Sustainable Perennial Crops Lab, USDA-ARS, Beltsville, MD USA; 3 Tropical Agriculture Research Station, USDA-ARS, Puerto Rico, PR USA

**Keywords:** Comparative genomics, Genome duplication, Genome evolution

## Abstract

Dragon fruits are tropical fruits economically important for agricultural industries. As members of the family of *Cactaceae*, they have evolved to adapt to the arid environment. Here we report the draft genome of *Hylocereus undatus*, commercially known as the white-fleshed dragon fruit. The chromosomal level genome assembly contains 11 longest scaffolds corresponding to the 11 chromosomes of *H. undatus*. Genome annotation of *H. undatus* found ~29,000 protein-coding genes, similar to *Carnegiea gigantea* (saguaro). Whole-genome duplication (WGD) analysis revealed a WGD event in the last common ancestor of *Cactaceae* followed by extensive genome rearrangements. The divergence time between *H. undatus* and *C. gigantea* was estimated to be 9.18 MYA. Functional enrichment analysis of orthologous gene clusters (OGCs) in six *Cactaceae* plants found significantly enriched OGCs in drought resistance. Fruit flavor-related functions were overrepresented in OGCs that are significantly expanded in *H. undatus*. The *H. undatus* draft genome also enabled the discovery of carbohydrate and plant cell wall-related functional enrichment in dragon fruits treated with trypsin for a longer storage time. Lastly, genes of the betacyanin (a red-violet pigment and antioxidant with a very high concentration in dragon fruits) biosynthetic pathway were found to be co-localized on a 12 Mb region of one chromosome. The consequence may be a higher efficiency of betacyanin biosynthesis, which will need experimental validation in the future. The *H. undatus* draft genome will be a great resource to study various cactus plants.

## Introduction

Dragon fruits (pitahaya or pitaya) are a group of *Hylocereus* species in the *Cactaceae* family. The genus *Hylocereus* was classified into 19 species based on morphological characteristics, and all of these species are indigenous to tropical America^1^. Among them, *Hylocereus undatus* (commonly known as the white-fleshed pitaya) is the most commonly cultivated species as a fruit crop^2^. The precise origin of *H. undatus* is not clear, but it is generally considered a native to Southern Mexico and Central America^3^. Today, this species has been commercially grown throughout tropical and subtropical regions of the world, particularly in Southeast Asia and Southern Mexico^4,5^.

In recent years, dragon fruits (including *H. undatus* and the red-fleshed *H. polyrhizus*) have been accepted as important fruit crops for their rich nutrient content^6,7^, including high amounts of antioxidants (e.g., betacyanin and other phenolics), dietary fibers, prebiotics, vitamins, and minerals (e.g., calcium and potassium). These nutrients have numerous benefits to human health, which may also explain the increased consumption of dragon fruit for health purposes^6,7^.

In Asia, dragon fruits are the fifth most popular tropical fruits, and the global production of *H. undatus* has been increasing. In Vietnam alone, there are 40,000 hectares (Ha) of land dedicated for dragon fruit production, which is worth ~$895 million annually^8^. In addition to being consumed as a fruit, *H. undatus* is also known as an ornamental plant owing to its exotic appearance and night flowering^7^. Also, its fruit rind or peel has a high content of betacyanins (one type of betalains), and therefore *H. undatus* has been used to produce coloring dyes and additives in the food and cosmetic industries^6,7^.

In addition to their significance in agricultural industries, as members of the family of *Cactaceae* dragon fruits are also important for the study of cactus plants in general. For example, species of *Cactaceae* are well known to be highly drought resistant. To adapt to an arid environment, dragon fruits and other cacti have developed fascinating structures, including succulent stems, acicular leaves, and watery fruits. In fact, the family *Cactaceae* arose ~28.8 (median with a range 15–45) millions of years ago (Mya)^9,10^, when the Earth underwent a drop in atmospheric CO_2_ concentration and a global expansion of aridity. As a result, plants in this family have gone through rapid genome evolution (e.g., whole-genome duplication and gene family expansion/contraction^11,12^) and species diversification, as well as drastic changes at the phenotypic level^13,14^, which leads to a conflict between molecular phylogeny and classification based on morphological characters^15,16^. Recently, five cacti genomes have been sequenced to study homoplasy among different cacti and its impact on molecular phylogenies^17^. Obviously, more genomes sequenced in *Cactaceae* will certainly further improve our understanding of the cacti evolution and adaptation to dry and hot climates.

Due to their economic importance, an increasing number of research papers have been published in recent years to study dragon fruits from the food science perspective^18–22^ and using RNA sequencing (RNA-seq) technology. For the latter, RNA-seq of *Hylocereus* species has been used to study abiotic stress response^23–25^, betalain synthetic pathway^26,27^, disease-resistant genes^28^, and antioxidant resistance during storage^29,30^. Due to the lack of a reference genome, all of these studies are based on mapping RNA-seq reads to de novo assembled transcripts instead of mapping to a reference genome.

In this study, we provide a high-quality genome assembly of *H. undatus* at the chromosome level. This highly continuous draft genome has allowed us to (i) study the whole-genome duplication of dragon fruits, (ii) date the divergence of *H. undatus* from other cacti, (iii) identify gene ontology functional groups that distinguish cacti from non-cacti plants, and distinguish *H. undatus* from other cacti, (iv) better understand the genomic adaptation of *H. undatus* to drought resistance, (v) study differentially expressed genes in trypsin-treated dragon fruit during storage by mapping RNA-seq reads to the *H. undatus* draft genome, and lastly, (vi) identify key enzymes that are involved in the synthesis of betacyanin, a red-violet pigment and antioxidant with a very high concentration in dragon fruits.

## Results

### The draft genome of *H. undatus* contains 11 long scaffolds at the chromosomal level

**Fig. 1:**
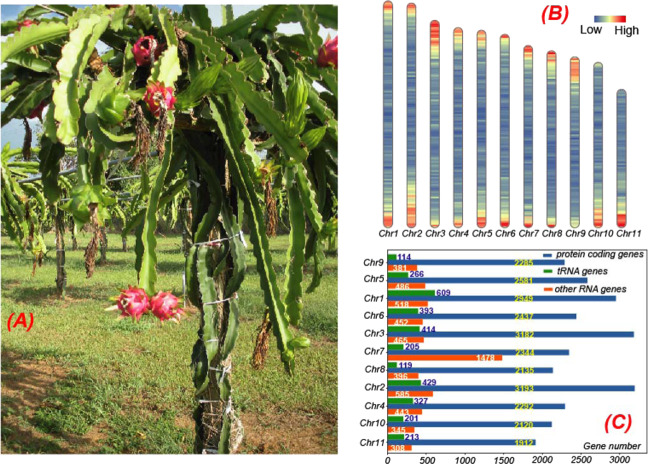
Gene distribution in the 11 longest scaffolds (pseudochromosomes) which account for 88.7% of the dragon fruit draft genome. Protein-coding genes, noncoding RNA gene, and tRNA genes resided in these scaffolds account for 87.8%, 72.6%, and 58.0% of all these genes, respectively. **A** A photo of the whole plant of *Hylocereus undatus* cultivar “David Bowie” from the USDA-ARS Tropical Agriculture Research Station in Mayaquez, Puerto Rico. **B** Protein-coding gene density of dragon fruit in the 11 longest scaffolds/pseudochromosomes with a window size 100,000 bp, which is plotted by Rldeogram^111^. **C** Distribution of protein-coding genes (blue), noncoding RNA genes (including rRNAs, orange), and tRNA genes (green) on the 11 longest scaffolds. The Chr7 (Scaffold 33675) has the most (1478) noncoding RNAs, including 1125 5S rRNAs. The mapping of scaffolds and pseudochromosomes is as follows: Chr1:Scaffold 33678, Chr2:Scaffold 19641, Chr3:Scaffold 33676, Chr4:Scaffold 10417, Chr5:Scaffold 33679, Chr6:Scaffold 33677, Chr7:Scaffold 33675, Chr8:Scaffold 33673, Chr9:Scaffold 33680, Chr10:Scaffold 3410, Chr11:Scaffold 2055

The genome of the *H. undatus* cultivar “David Bowie” (from Puerto Rico, Fig. 1A) was sequenced using a combination of 10X chromium sequencing, Chicago and Hi-C chromatin proximity ligation from Dovetail Genomics (see “Methods“ for details). The initial 10X raw read assembly had a contig N50 = 31 kb, and scaffold N50 = 769 kb. This assembly was significantly improved with Chicago and Hi-C data for scaffolding using the HiRise pipeline, which produced a much-improved assembly with a scaffold N50 = 109.7 Mb and assembly size = 1.33 Gb. Previous studies have indicated that the chromosome number of *H. undatus* is 2*n* = 22^31^, which has been further confirmed by flow cytometry^32–34^. Furthermore, the genome size of *H. undatus* was estimated to be 1.44 Gb^33^ with DNA 2C content = 3.63, using *Arabidopsis* as a reference (DNA 2C content = 0.32)^35^. Therefore, it is estimated that over 92.4% (1.33/1.44) of the *H. undatus* genome was assembled into 33,691 scaffolds. Interestingly, 88.7% of the assembled genome were present in the 11 longest scaffolds (Fig. 1B), corresponding to the 11 chromosomes reported in dragon fruit^32^. This suggests that our *H. undatus* genome assembly is close to the chromosomal level.

The completeness of the draft genome was further evaluated by BUSCO (Benchmarking Universal Single-Copy Orthologs)^36^. Of the 2121 single-copy orthologous genes in the BUSCO eudicotyledons_odb10 database, 1972 (93.0%) were identified in our draft genome (Supplementary Table S1). The distribution of genes (see below) in assembled scaffolds showed that almost all of these genes resided in the 11 longest scaffolds (Fig. 1C). A total of 89.1% RNA-seq reads, obtained from NCBI (SRR3234546^37^, sampled from the shoot of *H. polyrhizus* cultivar Zihonglong), were mapped to the draft genome. All these evaluations suggested high completeness and high continuity of the dragon fruit draft genome at the chromosome level.

### Genome annotation of *H. undatus* found ~29,000 protein-coding genes close to the number in *Carnegiea gigantea* (saguaro)

Repeat regions in the genome were annotated using a combination of ab initio and homology search methods (see “Methods”). As the result, 58.89% of the genome were identified as repeat regions (Supplementary Table S2). Long terminal repeat (LTR) elements were the largest category of classified/annotated repeats (22.47% of the genome). Most of the repeat regions (28.58%) were unclassified (Supplementary Table S2). For noncoding gene prediction, in total, 5637 tRNAs, 2364 rRNAs, and 5698 other noncoding RNAs were predicted in the dragon fruit draft genome (Fig. 1C).

For protein-coding gene prediction, MAKER^38^ was run with three iterations using RNA-seq and protein homology data as supporting evidence (see “Methods”). In total, 28,992 genes (length > 50 aa) were predicted (Table 1). Of the 28,992 proteins, 21,655 (74.7%) were annotated by eggNOG^39^, of which 10,093 were assigned to GO terms and 9920 were assigned to KEGG pathways.

**Table 1:** Statistics of assembly and annotation for six cactus plant draft genomes

Genome	Number of scaffolds	Scaffold N50	Assembly size (Mb)	Gene number (>=150 bp)	Average number of exons per gene	Average exon length (bp)	Average intergenic length (bp)^a^	Average protein length (aa)
Cgig	57,405	61.5 kb	980.4	28,288	5.35	243.93	7941.11	373.55
Hund	33,691	109 Mb	1332.5	28,992	5.29	263.44	35806.20	361.51
Lsch	158,705	9.3 kb	797.8	94,756	2.32	210.07	2521.97	205.03
Phum	126,352	4.4 kb	413.9	30,016	3.35	263.47	1960.61	294.75
Ppri	171,584	5.4 kb	629.3	41,825	3.43	230.01	2414.47	265.32
Sthu	159,478	10.5 kb	853.2	93,917	2.29	212.64	2807.56	203.95

*Hund* *Hylocereus undatus*, *Sthu* *Stenocereus thurberi*, *Lsch* *Lophocereus schottii*, *Cgig* *Carnegiea gigantea*, *Ppri* *Pachycereus pringlei*, *Phum* *Pereskia humboldtii*

^a^This is likely underestimated for all genomes except Hund due to the low N50 values (genomes are too fragmented)

In order to conduct a comparative genomic analysis, MAKER was also run to predict protein-coding genes in draft genomes of *Stenocereus thurberi, Lophocereus schottii, Pachycereus pringlei*, and *Pereskia humboldtii*, which were previously sequenced in ref. ^17^. The same previous paper also sequenced *Carnegiea gigantea* with a better genome assembly and the author has kindly provided us the predicted protein sequences, so MAKER prediction was not needed for this species. The statistics of protein-coding genes in the six cactus species (including *H. undatus*) are provided in Table 1. Notably, for *L. schottii* and *S. thurberi*, MAKER predicted 94,756 and 93,917 protein-coding genes, respectively. These numbers were three to four times larger than those of other cactus species, implicating possible whole-genome duplications (WGDs) in these two species (see more details below).

### Whole-genome duplication analysis predicts a WGD event in the last common ancestor of *Cactaceae* followed by extensive genome rearrangements in *H. undatus*

The large variation in the gene contents of the six cactus species (Table 1) demanded a whole-genome duplication (WGD) analysis. The paralogous and orthologous genes were identified by using a reciprocal best hit (RBH) approach with BlastP. The synonymous substitution rates (Ks) and the rate of transversions on fourfold degenerate synonymous sites (4dTv) of global paralogous genes within each species were calculated to infer potential WGD events. Similarly, the Ks and 4dTv rates of global orthologous genes between different species were also calculated. In addition to the six genomes of the family *Cactaceae*, we have also collected five sequenced genomes representing other families within the order of *Caryophyllales*: *Beta vulgaris* (Bv)^40^ and *Spinacia oleracea* (So)^41^ of *Chenopodiaceae*, *Aldrovanda vesiculosa* (Av) of *Droseraceae*^42^, *Simmondsia chinensis* (Sc) of *Simmondsiaceae*^43^, and *Fagopyrum tataricum* (Ft) of *Polygonaceae*^44^.

**Fig. 2:**
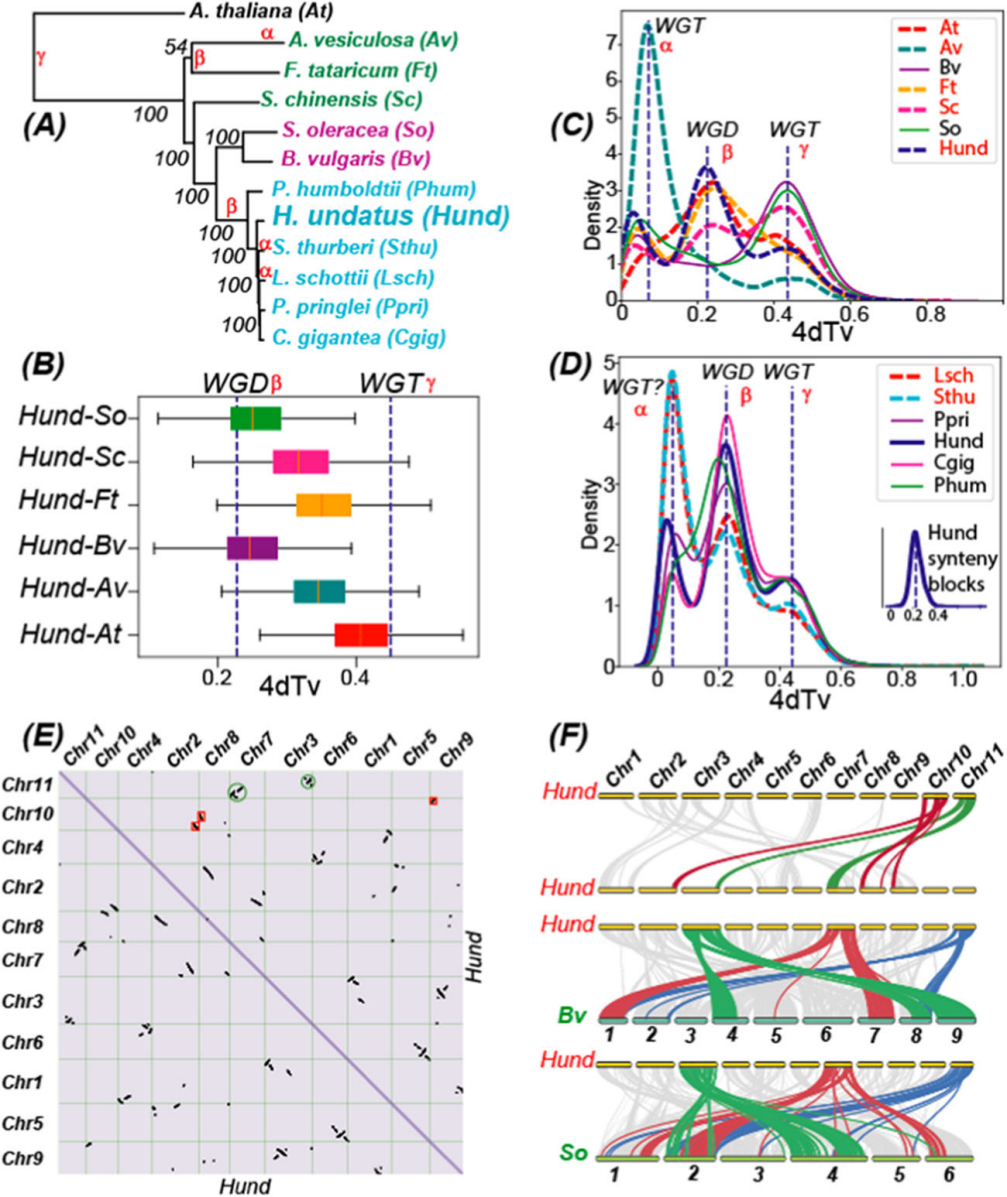
Whole-genome duplication (WGD) analysis of *Hylocereus undatus* (Hund) and 11 other species. The full names and acronyms of the 11 plants are: *Stenocereus thurberi* (Sthu), *Lophocereus schottii* (Lsch), *Carnegiea gigantea* (Cgig), *Pachycereus pringlei* (Ppri), and *Pereskia humboldtii* (Phum) of Cactaceae, *Beta vulgaris* (Bv)^40^, and *Spinacia oleracea* (So)^41^ of Chenopodiaceae, *Aldrovanda vesiculosa* (Av) of Droseraceae^42^, *Simmondsia chinensis* (Sc) of Simmondsiaceae^43^, and *Fagopyrum tataricum* (Ft) of Polygonaceae^44^, *Arabidopsis thaliana*. **A** A phylogeny is built with single-copy orthologous genes (identified by OrthoFinder) in the 12 genomes (concatenated alignment with MAFFT and tree built with RAxML). The WGD events characterized in (**C**) and (**D**) are indicated beside the branches. **B** Orthologous genes between Hund and other species were identified using the reciprocal best hit (RBH) approach, and fourfold degenerate synonymous sites (4dTv) values were calculated and plotted as boxplots. The dashed lines correspond to the WGD events characterized in (**C**). **C** Intragenome 4dTv distribution of paralogous genes (identified by RBH approach) in Hund and six other genomes. **D** Intragenome 4dTv distribution of paralogous genes (identified by RBH approach) in the six Cactaceae genomes. The inset figure shows the 4dTv distribution of only paralogous genes present in the synteny blocks (identified by MCScanX) of Hund. **E** Whole-genome self-alignment and syntenic blocks within the 11 largest Hund scaffolds/pseudochromosomes are presented as a dot plot. Strings of dots (paralogous genes) that correspond to duplicated regions in Chr11 (scaffold 2055) and Chr10 (scaffold 3410) are highlighted using the same circles or boxes in the same color. **F** A linear representation of the synteny analysis results is shown for intragenome Hund-Hund, as well as inter-genome Hund-Bv and Hund-So genome pairs. Synteny blocks of selected chromosomes are colored in red, green, and blue as examples. The chromosome sizes are drawn proportional to the actual genome size within each species but not between different species (total genome size: Hund (1.33 Gb), Bv (544 Mb), and So (969 Mb))

Using 507 single-copy genes identified in the 12 genomes (six *Cactaceae* + five other *Caryophyllales* + *Arabidopsis thaliana*), a phylogeny (Fig. 2A) was built to depict the phylogenetic relationship among these species. According to this phylogeny genomes of *Cactaceae* are closer to *Chenopodiaceae*, then to *Simmondsiaceae*, and lastly to *Droseraceae* and *Polygonaceae*. This is also supported by the 4dTv (Fig. 2B) and the Ks (Supplementary Fig. S1A) distributions of orthologous genes between Hund and the five *Caryophyllales* genomes.

The intragenome 4dTv distributions (Fig. 2C) of paralogous genes reveal three peaks in Hund. This is also true for two other *Caryophyllales* genomes (Ft and Sc) and the distant At. In contrast, Bv and So have two peaks. However, all the 12 genomes have the last peak (γ), which corresponds to the ancient whole-genome triplication (WGT) shared by all eudicot plants. The middle peak (β) is found in all species except for Bv and So, while its exact position differs slightly among different species. While this β peak is the largest peak in Hund, At, and Ft, it is very small and almost neglectable in Av. Remarkably, Av also distinguishes itself from other species with a very large first peak (α), which has been reported to be a very recent WGT event unique in Av^42^. In all other species, this α peak is much smaller and has very small 4dTv values, suggesting it might not represent a WGD but rather a smaller scale recent duplications such as tandem or dispersed duplications. The reason is that more recent WGD tends to result in larger peaks (e.g., the WGT in Av creates the largest α peak), as more recently duplicated genes have not degenerated as much as more anciently duplicated genes. We also provided the Ks distributions of the paralogous genes in Supplementary Fig. S1A, where the separation of the α and β peaks is not very evident in most species.

Further 4dTv analysis of the other five *Cactaceae* genomes (Fig. 2D), despite their low N50 assemblies, found that they all share the β peak and the γ peak with Hund. The positions of the two peaks are also fairly consistent in these six genomes. In contrast, the α peak differs significantly: it is the largest in Lsch and Sthu, very small in Phum (almost neglectable), and medium in Ppri, Hund, and Cgig. Considering that the gene numbers in Lsch and Sthu are at least three times larger than the other four genomes (Table 1), one can speculate that this α peak might correspond to a more recent WGT event that had only happened in Lsch and Sthu. However, this speculation needs a more vigorous study once the genome assemblies of the two species are much-improved. The current very fragmented assemblies of two genomes hinder the verification of the putative WGT in these genomes via a syntenic block analysis.

According to Fig. 2B, the β peak (first dashed line) has a lower 4dTv value than the Hund-Bv and Hund-So 4dTv values (box plot), suggesting that the WGD event happened after the divergence of *Cactaceae* from other *Caryophyllales* families. Similarly, the γ peak (second dashed line) has a higher 4dTv value than the Hund-At values (box plot), confirming that the ancient WGT event happened before the emergence of the common ancestor of Hund and At, i.e., at the base of all dicot plants. To approximately estimate the absolute dates of the three WGD events, we used the formula: *T* = *Ks/2γ*, where γ = 1.5 × 10^−8^ is the rate of synonymous substitutions per site per year for dicots following^45^. From the Ks distributions of the paralogous genes in the six *Cactaceae* (Supplementary Fig. S2B), we estimated that the α peak (WGT in Lsch and Sthu) corresponds to 6.0 MYA, the β peak (WGD in the last common ancestor of *Cactaceae*) corresponds to 25.7 MYA, and the γ peak (the ancient core dicot WGT) corresponds to 135.0 MYA.

With the chromosome-level assembly of Hund, we have also performed a whole-genome self-alignment (Fig. 2E), an intragenome syntenic block analysis (Fig. 2F), as well as an inter-genome syntenic block analysis (Fig. 2F) of Hund vs. the previously published chromosome-level assemblies of Bv^40^ and So^41^. These results show that dragon fruit had experienced a WGD event followed by extensive genome rearrangements (e.g., chromosome breakage and reorganization) (Fig. 2E). For example, Chr11 (Scaffold 2055) shares large syntenic gene blocks with Chr8 (Scaffold 33675) and Chr3 (Scaffold 33676), while Chr10 (Scaffold 3410) shares large syntenic gene blocks with three chromosomes: Chr2, Chr8, and Chr9. It also appears that the genomic synteny is fairly conserved between *Cactaceae* and *Chenopodiaceae* genomes according to the inter-genome Hund-Bv and Hund-So synteny alignments (Fig. 2F). The inter-genome alignments of Hund-Bv and Hund-So were also made (Supplementary Fig. S8A, B). These alignments show that there is a clear two-to-one relationship between Hund and the other two genomes with respect to the aligned syntenic blocks, which supports the lack of the β WGD peak in Bv and So (Fig. 2C).

Notably, Bv and So are among the earliest sequenced *Caryophyllales* genomes and both have high-quality chromosome-level assemblies. The chromosome-level assembly of Bv has been achieved by genetic and physical mapping as well as classic BAC/Fosmid clones and long-read Sanger/454 sequencing^40^. The chromosome-level assembly of So has been supported by BioNano optical mapping data and long-range mate-pair sequencing libraries^41^. Therefore, the inter-genome synteny alignments shown in Fig. 2F also suggest that our chromosome-level assembly of Hund genome is of high quality. In contrast, we were not able to perform the intragenome and inter-genome syntenic block analyses for the other five cacti because their genome assemblies have much lower continuity (low N50 and too many contigs) (Table 1).

To study which of the three 4dTv peaks in Fig. 2D correspond to the Hund intragenome syntenic blocks plotted in Fig. 2E, F, we have extracted the paralogous genes located in the syntenic blocks only and plotted their 4dTv distribution. The inset plot of Fig. 2F clearly shows that these Hund syntenic blocks correspond to the β peak in Fig. 2D. This again supports that the small α peak in Hund is not derived from a WGD; otherwise, the 4dTv distribution of Hund syntenic blocks will have a peak corresponding to the α peak. Furthermore, the Ks distributions of the six *Cactaceae* genomes (Supplementary Fig. S2B) also reveal that the α peak is only evident in Hund, Lsch, and Sthu, and it is much smaller than the β peak in Hund. Overall, all the evidence suggests that unlike Lsch and Sthu, Hund does not have a very recent WGD after its divergence from other *Cactaceae* species.

To further study this small α peak of Hund in Fig. 2D, we have extracted 2,250 Hund genes with 4dTv < = 0.116 (the entire α peak) and performed Pfam (Supplementary Table S3) and GO (Supplementary Table S4) annotation on them. Interestingly, 632 (28%) of these 2250 genes are located within 10 genes from each other, indicating that these genes were derived from tandem duplications. The Pfam annotation showed that the most abundant protein families include plant transposases, protein kinases, plant disease resistance proteins (leucine-rich repeat), cytochrome P450, and 2OG-Fe(II) oxygenases (Supplementary Table S3). All these protein families might have been selected in Hund to have tandem gene duplications as a genomic adaptation to the hot and dry environments.

### The last common ancestor of *H. undatus* and *C. gigantea* is estimated to appear 9.18 MYA

**Fig. 3:**
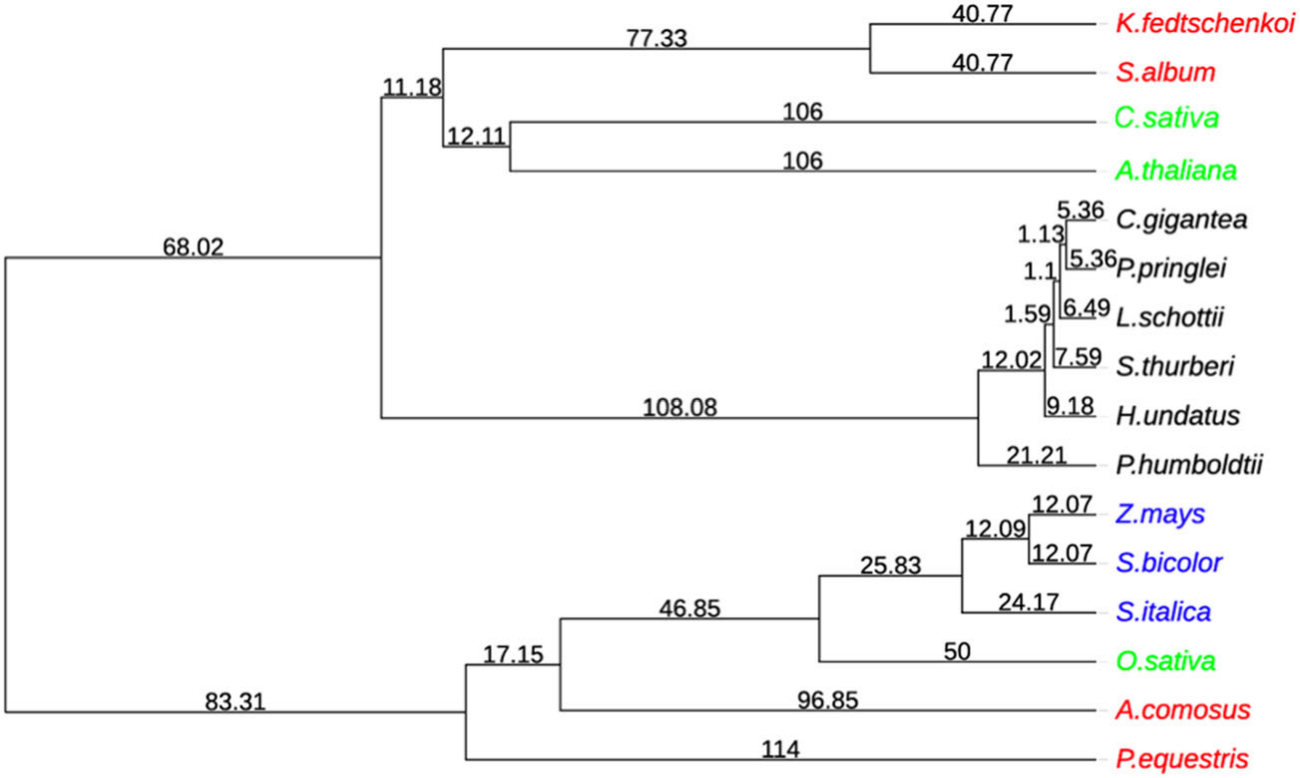
Species tree and divergence times of 16 plant species. CAM plants are colored in red (noncactus family) and black (cactus family). C3 and C4 are colored in green and blue, respectively. The maximum-likelihood tree was built with 130 single-copy genes using RAxML. The divergence time is estimated by r8s. The full names of the 16 plants are: three C3 plants (*Oryza sativa*^46^, *Arabidopsis thaliana*^47^, and *Cannabis sativa*^48^), three C4 plants (*Zea mays*^49^, *Sorghum bicolor*^50^, and *Setaria italica*^51^), four CAM plants (*Phalaenopsis equestris*^52^, *Ananas comosus*^53^, *Kalanchoe fedtschenkoi*^54^, and *Sedum album*^55^), and six cactus plants (*Hylocereus undatus*, *Stenocereus thurberi*^17^, *Lophocereus schottii*^17^, *Carnegiea gigantea*^17^, *Pachycereus pringlei*^17^, and *Pereskia humboldtii*^17^)

To examine the evolutionary relationship within the six cactus plants and between cactus plants and other plants, we have included ten additional plant genomes in our analyses. These include three C3 plants (*Oryza sativa*^46^*, Arabidopsis thaliana*^47^, and *Cannabis sativa*^48^), three C4 plants (*Zea mays*^49^*, Sorghum bicolor*^50^, and *Setaria italica*^51^), and four CAM (crassulacean acid metabolism) plants (*Phalaenopsis equestris*^52^*, Ananas comosus*^53^*, Kalanchoe fedtschenkoi*^54^, and *Sedum album*^55^). Using OrthoFinder^56^, 645,270 proteins of these 16 plant genomes were clustered into 31,276 orthologous gene clusters (OGCs) with each cluster containing more than two proteins. A phylogenetic tree was built using 130 single-copy genes to represent the species tree (Fig. 3). The topology of the grass family agrees with the previous analysis^57^, and the topology of the cactus family is also in line with the cactus phylogeny described in ref. ^17^, suggesting the high quality of this species tree.

With the species tree, r8s^58^ was employed to estimate the divergence time of different cactus plants. Three divergence times from the TimeTree database^59^ were used as references: (i) 50 MYA (million years ago) between *O. sativa* and *S. bicolor*, (ii) 106 MYA between *C. sativa* and *A. thaliana*, and (iii) 114 MYA between *A. comosus* and *P. equestris*. Shown in Fig. 3, the divergence times of cactus family species were estimated to be: (i) 5.36 MYA between *C. gigantea* and *P. pringlei*, (ii) 6.49 MYA between *C. gigantea* and *L. schottii*, (iii) 7.59 MYA beween *C. gigantea* and *S. thurberi*, (iv) 9.18 MYA between *C. gigantea* and *H. undatus*, and (v) 21.12 MYA between *C. gigantea* and *P. humboldtii*. As expected, the estimated time of *P. humboldtii* diverging from other cacti (21.12 MYA) was more recent than the estimations made for the cactus crown clade in ref. ^17^ (26.88 MYA) and in ref. ^60^ (30–30.5 MYA).

### Functional enrichment analysis of orthologous gene clusters (OGCs) in cactus plants finds significantly enriched OGCs in drought resistance

**Fig. 4:**
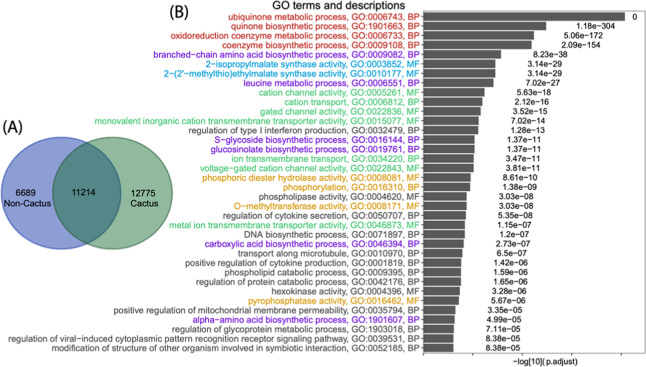
GO enrichment analysis of orthologous gene clusters (OGCs) in cactus and noncactus plants. **A** 30,457 OGCs are clustered into 12,755 cactus-specific OGCs, 6689 noncactus-specific OGCs, and 11,214 shared OGCs. **B** GO enrichment analysis was conducted with 12,775 cactus-specific OGCs as foreground, and 11,214 shared OGCs as background. The *x* axis shows the log10 of the adjusted *P* values, and the *y* axis shows the GO terms (only molecular function (MP) and biological process (BP) are shown) with adjusted *P* value < 0.01. Groups of GO terms are colored in green (group I), red (group II), purple (group III), blue (group IV), and orange (group V). See main text for the five groups of GO terms

Of these 31,276 OGCs, 30,678 contain proteins from more than two species. We have performed gene ontology (GO) enrichment analysis between OGCs of different groups of plants following the method in our previous paper^61^. The goal was to investigate, comparing to the shared OGCs (as control), what GO functional terms are significantly overrepresented in the cactus-specific OGCs. These enriched GO terms may highlight what biological functions are critical for cacti to adapt to their unique living environments. Briefly, the 30,678 OGCs were separated into three groups according to what species are present in the OGCs (Fig. 4A). A cactus-specific OGC was defined as a cluster containing proteins from at least two cactus species but not from any noncactus species. A shared OGC contained at least one cactus and one noncactus species. Then for each GO term, a binomial test *P* value was calculated to measure the statistical enrichment of this GO term in cactus-specific OGCs, by considering the count of cactus-specific OGCs and the count of shared OGCs assigned to this GO.

Comparing to noncactus plants, cactus plants can survive in arid environments with high light, hot temperature extremes, and little water. We anticipated that the GO enrichment analysis could reveal functions that can help explain these traits unique in cactus plants. Indeed, among the list of significantly enriched GO terms shown in Fig. 4B, 23 (63.8%) can be classified into five groups of functions that are related to cactus adapting to the dry and hot environments.

1. Group I (green) contains GO terms related to ion-channel functions. This is expected as they are highly related to osmotic stress and controlling movements of the stoma. For example, during the daytime, cactus plants (and all CAM species) close their stoma to reduce water transpiration.

2. Group II (red) is antioxidant defense-related. To survive in an arid environment, cactus plants must possess the drought response mechanism^62^, particularly the antioxidant defense system. The constant drought stress will lead to the accumulation of the oxidizing substance, such as O_2_, H_2_O_2_, O_2_-, and OH, which will damage the cells and even cause the death^62^. Hence, antioxidant defense system-related GO terms are enriched in cactus plant genomes.

3. Group III (purple) is related to biosynthetic metabolism, particularly amino acid biosynthesis. Evidence has been shown in model plant organisms that drought stress can induce alterations in almost all primary metabolisms^63^ including carbohydrates (e.g., glycosides), amino acids (e.g., branched-chain amino acids^64^), and lipids, as well as secondary metabolisms (e.g., glucosinolate^65^). Hence, these enriched GO terms can be associated with the drought resistance response of cactus plants as well.

4. Group IV (blue) contains GO:0003852 (2-isopropylmalate synthase activity) and GO:0010177 (2-(2’-methylthio) ethylmalate synthase activity), which are related to the CAM pathway. Therefore, these enriched functions should contribute to the CAM photosynthesis in cactus plants to conserve water and adapt to arid environments.

5. Group V (orange) is highly related to phosphoryl and methyl metabolism. *O*-methyltransferase is related to the biosynthesis of flavonoid, one of the most abundant classes of plant secondary metabolites^66^. All the other GO terms in this group are related to phosphorylation of proteins, carbohydrates, and lipids, which are critical responses to abiotic stresses including heat and drought^67^.

Similar enrichment analysis was also conducted on KEGG pathways (Supplementary Fig. S2). The enriched KEGG terms were classified into two groups: one contains pathways involved in environmental information processing and signal transduction, and the other contains pathways for the metabolism of various molecules (carbohydrates, proteins, lipids, glucosinolate, 2-oxocarboxylic acid, terpenoids, and polyketides). The first KEGG-term group corresponds to enriched GO group I and V, while the second group corresponds to enriched GO group II, III, and IV. Overall, the KEGG enrichment result is consistent with the GO enrichment result.

### Fruit flavor-related GO terms are found to be enriched in OGCs significantly expanded in *H. undatus*

**Fig. 5:**
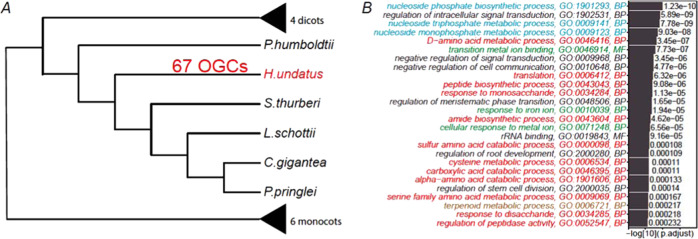
GO enrichment of significantly expanded OGCs in *H. undatus*. **A** The 67 significantly expanded OGCs were identified using CAFE with the species tree and OGCs generated by OrthoFinder as input. The complete version of the tree and OGCs on each node can be found in Supplementary Fig. S3. **B** Significantly enriched GO terms in the 67 significantly expanded OGCs in *H. undatus*. The full names of the 16 plants are provided in the legend of Fig. 3

In addition to the GO enrichment analysis performed on cactus-specific OGCs, we have also taken advantage of the species tree reconstructed from the 130 single-copy OGCs to identify OGCs significantly expanded along with specific nodes in the tree (Fig. 5A and Supplementary Fig. S3). The significantly contracted and expanded OGCs on different nodes in the tree were determined by CAFE^68^. Particularly, we have focused on the 67 OGCs that are significantly expanded in only dragon fruit (Fig. 5A).

The 67 OGCs were used as the foreground for statistical analysis. For background, we have selected conserved OGCs that contain at least one species from each of the three major clades of the species tree: cactus clade (six species), other dicot clade (four species), and monocot clade (six species). Then, for each GO term in the foreground, the *P* value of the binomial test was calculated to measure the significance of the GO enrichment.

Most enriched GO terms in these 67 OGCs were related to metabolisms of saccharide, amino acid, and carboxylic acid (colored in red in Fig. 5B) (gene and GO list in Supplementary Table S5). Sugar and acid metabolism processes have been shown to be related to the fruit flavor^69–72^. Therefore, OGCs significantly expanded in *H. undatus* have likely contributed to the dragon fruit maturity and ripening or its unique nutrition and flavor. In addition, evidence has also been shown that soluble sugars^73^ and other primary metabolites^63^ confer tolerance to drought stress. Other enriched GO terms include metal ion response functions (green in Fig. 5B), nucleoside and ribonucleotide metabolic processes (blue in Fig. 5B), terpenoid metabolic processes (brown in Fig. 5B), which are all related to drought resistance and fruit flavor as well^74,75^.

### Plant cell wall-related functions are enriched in differentially expressed genes (DEGs) in trypsin-treated dragon fruit during storage

Recent work has revealed that antioxidant functions were enriched in the differentially expressed genes (DEGs) between dragon fruit peels treated with and without trypsin^30^. The analysis was based on the de novo assembled transcripts without a reference genome. Hence, in this study, we mapped the RNA-seq reads to the dragon fruit draft genome for a more accurate reference-based DEG analysis. Briefly, the trypsin-treated (SRR8327215, *H. undatus* cultivar Viet 1 peel) and control (SRR8327214, *H. undatus* cultivar Viet 1 peel) clean reads were aligned to the *H. undatus* draft genome. After a reference-based transcript assembly, transcripts corresponding to 18,808 MAKER predicted genes (Supplementary Table S6) were analyzed by DESeq2^76^ for DEGs. As the result, 1065 significantly upregulated genes (*P*.adjusted < 0.05, log2FoldChange > 1) and 1279 significantly downregulated genes (*P*.adjusted < 0.05, log2FoldChange < −1) were identified and subject to GO enrichment analysis with all the 18,808 expressed genes as the background.

**Fig. 6:**
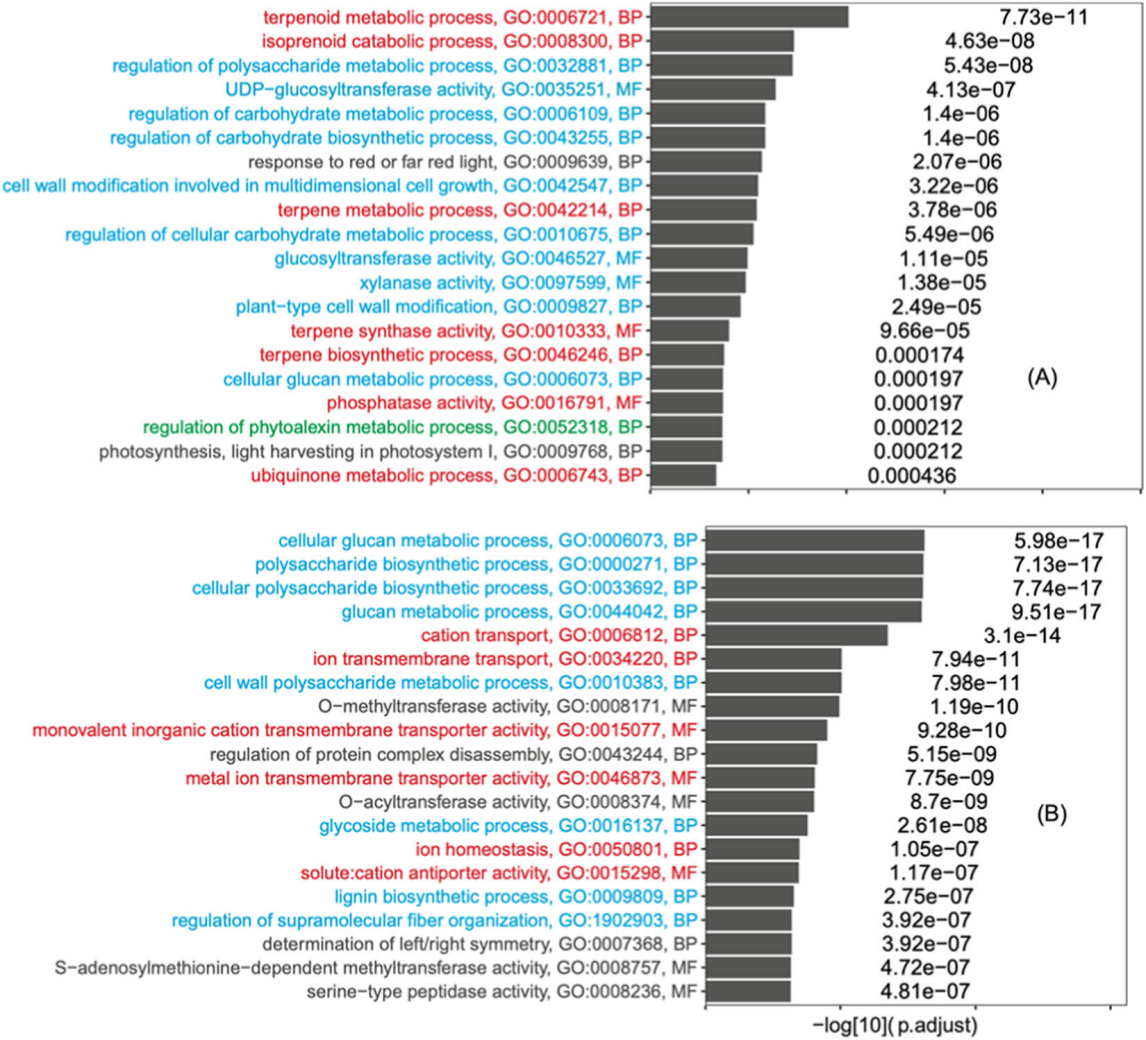
Top 20 GO terms enriched in DEGs. **A** Upregulated DEGs with trypsin treatment: superoxide scavenging activity-related GO terms are colored in red; saccharide-related GO terms are colored in blue; Phytoalexin is colored in green. **B** Downregulated DEGs with trypsin treatment: ion-related GO terms are colored in red; saccharide-related GO terms are colored in blue

As shown in Fig. 6A, the enriched GO terms in upregulated DEGs were classified into groups and colored differently. Group I (red) contained five terpenoid metabolic GO terms, which are antioxidants-related^77,78^ and consistent with the previous DEGs analysis based on de novo assembled transcripts^30,79^. Very interestingly, ten GO terms of group II (blue in Fig. 6A) contained various carbohydrate and plant cell wall-related processes, particularly those regulating functions. This was not found in ref. ^30^ but was expected as trypsin treatment may indirectly affect the cell wall integrity and carbohydrate metabolisms by directly acting on cell wall proteins. In addition, there were also other GO terms that respond to various biotic and abiotic stresses, such as regulation of phytoalexin metabolic process, ubiquinone metabolic process, phosphatase activity, and photosynthesis-related processes. The downregulated DEGs were also enriched with eight carbohydrate and plant cell wall-related processes (particularly those biosynthetic functions, blue in Fig. 6B), which is also the largest GO group. The other GO groups were related to ion transporting activities, which was found previously^30^. Overall, the genome reference-based DEG analysis revealed many more enriched GO terms than the previous DEG analysis based on de novo assembled transcripts.

### Most betalain biosynthetic genes are co-localized in a single *H. undatus* chromosome

**Fig. 7:**
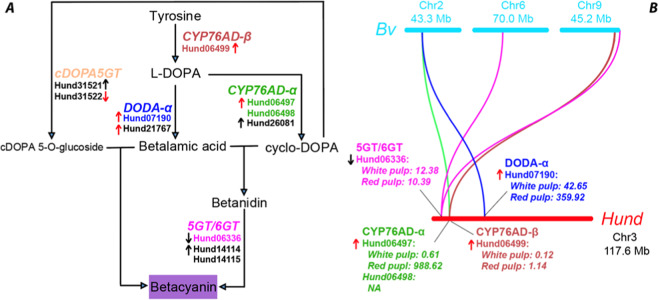
The betacyanin biosynthetic pathway in *H. undatus* and *B. vulgaris*. In (**A**), the enzyme subfamilies (larger font) and the *H. undatus* orthologous genes (smaller font) are shown. The up arrows show increased gene expressions from white pulp to red pulp development stages; the down arrows mean decreased expressions. The red arrows indicate significant expression changes (adjusted *P* value <0.05 and expression log2 (fold change) >1 or <−1), while black arrows do not. The colored genes are genes that are co-localized in Chr3 of *H. undatus*. In (**B**), the colors of genes and curved lines (depicting orthologous gene relationships) match the colors of genes in (**A**). The TPM (transcripts per kilobase million reads) values are also indicated for these co-localized genes on Chr3. The detailed expression values can be found in Supplementary Table S8

Betalains are red-violet (betacyanin) and yellow (betaxanthin) pigments uniquely found in the order *Caryophyllales*. The betacyanin synthesis pathway is illustrated in Fig. 7A, where the enzymes and their orthologs found in *H. undatus* genome are indicated. Betaxanthin is made from betalamic acid without enzymes by spontaneously connecting with amino acids, which is not shown in Fig. 7A. Two key enzyme families, L‐DOPA 4,5‐dioxygenase (DODA) and cytochrome P450 enzyme CYP76AD, in the betalain synthesis pathway, had been phylogenetically studied in *Caryophyllales*^80^. As a result, two DODA subfamilies (DODA-α and DODA-β) and three CYP76AD subfamilies (CYP76AD-α, CYP76AD-β, and CYP76AD-γ) were characterized. Only DODA-α, CYP76AD-α, and CYP76AD-β have been shown to be involved in the betalain synthesis pathway. Two glucosyltransferases (cyclo‐DOPA 5‐*O*‐glucosyltransferase [cDOPA5GT] and betanidin glucosyl‐transferase [5GT/6GT]) of glycosyltransferase family 1 (GT1), which are involved in structural modifications of betalains, were also more recently investigated^81^.

A homology search of the three enzyme families (DODA, CYP76AD, and GT1) followed by detailed phylogenetic analyses found that all of them have orthologs in the *H. undatus* genome (Fig. 7A and Supplementary Table S7). Specifically, Hund06497, Hund06498, and Hund26081 are the orthologs of CYP76AD-α, Hund06499 is the ortholog of CYP76AD-β, Hund27099, and Hund27100 are the orthologs of CYP76AD-γ (Supplementary Fig. S4). In the dragon fruit genome, CYP76AD genes seem to have many recently duplicated copies; these genes have a low Ks <0.3 (see above) and are located adjacently in the genome, suggesting a significant tandem duplication of CYP76AD in the dragon fruit genome. Similarly, for DODA enzymes, Hund07190 and Hund21767 are the orthologs of DODA-α; Hund21766 is the ortholog of DODA-β (Supplementary Fig. S5). Note that Hund21767 is located next to Hund21766 in the genome and shares 48.3% sequence identity. Lastly, Hund06336 is the ortholog of betanidin 5GT, Hund14114 and Hund14115 are the orthologs of betanidin 6GT, and Hund31521 and Hund31522 are the orthologs of cDOPA5GT (Supplementary Fig. S6).

Interestingly, Hund06336 (5GT), Hund06497 (CYP76AD-α), Hund06498 (CYP76AD-α), Hund06499 (CYP76AD-β), and Hund07190 (DODA-α) are all located in a ~12 Mb region of the Chr3 (Scaffold 33676) (total length 117.6 Mb) (Fig. 7B). Therefore, all the major genes in the betacyanin biosynthetic pathway except for cDOPA5GT are co-localized in one single chromosome of *H. undatus*. All these genes were also found in the closely related *H. polyrhizus* (Supplementary Table S7), which currently only have transcriptomes. As a comparison, in *Beta vulgaris*, these genes are located on three different chromosomes (Fig. 7B), although two key genes of them (CYP76AD-α and DODA-α) have been known to be adjacent to each other in one gene cluster in *B. vulgaris* and representative genomes of Amaranthaceae family^82^. We also attempted to locate these genes in the *C. gigantea* genome. However, as it is very fragmented compared to *H. undatus* and *B. vulgaris*, all these *C. gigantea* genes are found on different short contigs and thus it is not possible to determine if they are also clustered in *C. gigantea* genome.

A recent study has generated RNA-seq data in *H. polyrhizus* aiming to identify differentially expressed genes in different stages of dragon fruit pulp development (white pulp vs. red pulp)^27^. We have mapped ~12 Gb reads of this dataset to our *H. undatus* genome and found that 93% of the reads were mapped. Interestingly, 9 out of the 11 betacyanin biosynthetic genes in Fig. 7A have expression changes, among them, four have significantly increased expression changes (*P* value < 0.05 and log2 (fold change) > 1) from white pulp to red pulp development. Particularly, Hund06497 (CYP76AD-α) is the third most upregulated gene with a log2(fold change) = 10.2 among all Hund genes, followed by Hund07190 (DODA-α) with a log2 (fold change) = 2.6 (Fig. 7B and Supplementary Table S8).

Interestingly, crassulacean acid metabolism (CAM) genes are also differentially expressed in dragon fruit development. The CAM pathway genes were identified by using a list of query proteins (11 families) from *Kalanchoe fedtschenkoi*^54^ for a BlastP search in *H. undatus* followed by detailed phylogenetic analyses. As expected, all the main enzymes in the CAM pathway were found in *H. undatus*. Interestingly, among 38 *H. undatus* genes of the 11 enzyme families (Supplementary Table S9), 34 genes are differentially expressed (*P* value < 0.05) from white pulp to red pulp in dragon fruit development^27^. Ten of these genes are significantly upregulated (log2 (fold change) >1) and nine are significantly downregulated (log2 (fold change) < −1). The increased gene expression is especially evident for enzymes active in daytime. What this implicates will need further in-depth studies in the future.

## Discussion

Dragon fruits contain many species of the genus *Hylocereus* and are increasingly important for food and agricultural industries. In this study, we have sequenced the *H. undatus* draft genome, the first genome of the *Hylocereus* genus. This genome is also the sixth sequenced genome of the *Cactaceae* family. Compared to the previously published five *Cactaceae* genomes (*S. thurberi (Sthu), L. schottii (Lsch), C. gigantea (Cgig), P. pringlei (Ppri)*, and *P. humboldtii (Phum)*), the *H. undatus* genome has the longest scaffold N50 (109 M vs 61.5 k of *C. gigantea*, the best assembled in ref. ^17^, i.e., >1700 times longer) and the largest genome completeness (92.4% vs. 75.3% of *C. gigantea*).

The chromosome-level assembly of *H. undatus* genome is achieved by using the Dovetail Genomics HiRise™ scaffolding software with long-range Chicago libraries and Hi-C libraries sequencing. This pipeline has been widely used by a number of large plant and animal genome-sequencing projects^83–86^. Evaluation of its performance by resequencing reference genomes of model organisms such as humans has demonstrated that it is an accurate and inexpensive approach to building long-range sequence scaffolds at the chromosome-level even^86–88^. The density report in the Hi-C scaffolding run on our *H. undatus* genome shows that the Hi-C data is in agreement with the placement and orientation of the input scaffolds in the final chromosome-level assembly (Supplementary Fig. S7). In addition, the inter-genome synteny alignments of *H. undatus* against four other chromosome-level genomes of *Caryophyllales* (Fig. 2F and Supplementary Fig. S8) also suggest that our *H. undatus* genome assembly is in a high quality.

With this high-quality draft genome, comparative analysis against other cacti genomes revealed a number of interesting findings as described in “Results”. These findings have led to new understandings ranging from whole-genome duplication, species divergence time, to significantly enriched GO functions in *Cactaceae* and in *H. undatus*, as well as betacyanin biosynthetic genes co-localized in a 12 Mb region of one single chromosome.

Particularly, the highly continuous chromosome-level assembly of *H. undatus* genome has allowed us to detect and confidently verify the WGD in *H. undatus*. This WGD event (Fig. 2) happened most likely in the last common ancestor of all cactus plants (β node in Fig. 2A). This is because the 4dTv β peak is clearly shared by all the six studied cacti. In addition, it is obvious that *S. thurberi* and *L. schottii* have had an extra recent WGT event (α peak in Fig. 2D). This observation is also consistent with the peculiarly higher numbers of protein-coding genes predicted from *S. thurberi* and *L. schottii* draft genomes (Table 1).

Similarly, it is the chromosome-level *H. undatus* genome that made it possible to locate the betacyanin pathway genes within a 12-Mb genomic region of the Chr3 (Fig. 7B). In contrast, although orthologous genes were also identified in *C. gigantea* and *H. polyrhizus*, it is unknown if these genes are also co-localized in other cactus genomes. The future improvement of these fragmented draft genome to a chromosome-level assembly will help to answer this question. It is interesting to note that although CYP76AD-α and DODA-α genes are adjacent in Chr2 of the well-assembled *B. vulgaris* genome, other core genes in the pathway are located on two other chromosomes (Chr6 and Chr9) (Fig. 7B). The chromosome-level co-localization of betacyanin biosynthetic genes in *H. undatus* may implicate a more efficient biosynthesis of betacyanin. The experimental verification of this hypothesis in the future is very necessary, given that betacyanin is one type of highly active and abundant antioxidants found in many cactus plants.

Lastly, it is widely accepted that reference-based RNA-seq assembly and expression quantification are preferred whenever a reference genome is available^89^. This is because de novo transcript assembly tends to have various issues (e.g., more fragmented and redundant contigs, missing low-abundance transcripts, mis-assembled transcripts), which may lead to inaccurate or incomplete results in downstream differential expression analysis. Indeed, by using the available *H. undatus* draft genome as the reference, we have identified antioxidant-related GO terms enriched in differentially expressed genes (DEGs) in trypsin-treated dragon fruits, which confirmed the results in refs. ^30,79^. Furthermore, we were also able to identify carbohydrate and plant cell wall-related biological processes to be also highly enriched in DEGs.

In summary, the *H. undatus* draft genome will be a significant contribution to the dragon fruit research community. With a chromosome-level assembly and high genome completeness, it will also be a great reference for the study of various cactus plants. As dragon fruits are increasingly consumed as an important tropical fruit, various dragon fruit cultivars have been developed all over the world. This *H. undatus* draft genome will be a great resource to develop genomic tools such as SNPs and other molecular markers for genetic characterization of various dragon fruit cultivars.

## Materials and methods

The detailed methods can be found in the Supplementary Method file. Here, we only briefly describe the materials and methods used in this study.

### Plant material, 10X library prep, and sequencing

Stem (cladode) samples of *H. undatus* cultivar “David Bowie” (Fig. 1A) were collected from the USDA-ARS Tropical Agriculture Research Station in Mayaquez, Puerto Rico. Whole-genome sequencing libraries were prepared using Chromium Genome Library & Gel Bead Kit v.2 (10X Genomics, cat. no. 120258) and sequenced on a NovaSeq6000 sequencer (Illumina, San Diego, CA) with paired-end 150 bp reads.

### Chicago library preparation and sequencing

Two Chicago libraries were prepared as described previously^88^. The libraries were sequenced on an Illumina HiSeq X. The number and length of read pairs produced for each library were: 145 million, 2 × 150 bp for library 1; 181 million, 2 × 150 bp for library 2. Together, these Chicago library reads provided 235.75× physical coverage of the genome (1–100 kb pairs).

### Dovetail Hi-C library preparation and sequencing

Two Dovetail Hi-C libraries were prepared in a similar manner as described previously^88^. The libraries were sequenced on an Illumina HiSeq X. The number and length of read pairs produced for each library were: 204 million, 2 × 150 bp for library 1; 177 million, 2 × 150 bp for library 2. Together, these Dovetail Hi-C library reads provided 6,171.67× physical coverage of the genome (10–10,000 kb pairs).

### Scaffolding the assembly with HiRise

The input de novo assembly, shotgun reads, Chicago library reads, and Dovetail Hi-C library reads were used as input data for HiRise, a software pipeline designed specifically for using proximity ligation data to scaffold genome assemblies^90^. An iterative analysis was conducted.

### Repeat and noncoding RNA annotation

RepeatModeler^91^ and RepeatMasker^92^ were employed to annotate repetitive elements in the draft dragon fruit genome and other cactus genomes. tRNA-scan2^93^ was used to identify tRNA genes. Infernal package^94^ and Rfam^95^ were employed to identify noncoding RNA genes.

### Protein-coding gene prediction

MAKER^38^ was employed to predict protein-coding genes by combining ab initio and homology-based approaches. Three rounds of MAKER runs considered transcriptome and protein evidence as well as ab initio gene prediction. The result of the third round of MAKER run was used as the final protein-coding gene model. In addition to *H. undatus*, MAKER gene predictions were also performed on four of the five cactus draft genomes sequenced by^17^ except for *C. gigantea* following the same procedure as described above for *H. undatus*. The redundant proteins from MAKER predictions were removed using seqkit^96^, so were proteins < 50 aa.

### Orthologous gene clusters and phylogenetic analyses

Proteins of a total of 16 sequenced plant genomes were selected to define orthologous gene clusters (OGCs) and for phylogenetic analyses. These genomes include three C3 plants, three C4 plants, and ten CAM plants. Six of the ten CAM plants are cacti, and their proteins were obtained by the processes described above. Some genomes have proteins from alternative splicing, and such genomes were processed to only keep the longest isoform protein of each gene.

Proteins of the 16 genomes were combined as input to OrthoFinder^56^. All of the single-copy orthologs were aligned with MUSCLE^97^. The alignments of single-copy OGCs were concatenated into one super alignment, which was further processed by Gblocks^98^. A phylogenetic tree was built using RAxML^99^ to represent the species tree with 100 times of bootstrap and the evolutionary model -m PROTGAMMAJTT. The divergence time of the 16 plants was estimated by r8s^58^ with three calibrations. The input tree to r8s was the tree built by RAxML. All the OGCs generated by OrthoFinder were analyzed by CAFE^68^ to identify significantly expanded and contracted OGCs in different nodes of the species tree.

### GO and KEGG enrichment analysis

OGCs generated by OrthoFinder were classified into different groups according to what plant species that a CGC contains proteins from. For GO and KEGG annotation, all the proteins within a CGC were BlastP searched against the eggNOG database^39^. The eggNOG hit contains GO term and KEGG term, which were transferred to the query protein. For each CGC, duplicated GO terms or KEGG terms were only counted once for the enrichment analysis. See “Results” for how we have defined foreground and background datasets depending on what questions we wanted to address. The same approach had been used in our previous papers^61,100^.

### Whole-genome duplication analysis

To examine the WGD in cactus species, wgd^101^ and MCScanX^102^ were used (unless stated otherwise) to analyze the synteny and calculate the synonymous substitution rate (Ks) and the rate of transversions on fourfold degenerate synonymous sites (4dTv).

### RNA-seq data analysis

For the expression analysis of trypsin-treated dragon fruit during storage, the control (SRR8327214) and trypsin-treated (SRR8327215) raw reads were downloaded from the NCBI SRA database. For the expression analysis of betacyanin biosynthetic genes and CAM genes, the white pulp (SRR2924904) and red pulp (SRR3203780) raw reads were downloaded from the NCBI SRA database. The reference genome-based differential expression analysis used HISAT2^103^ and StringTie pipeline following^104^. DEseq2^76^ was used to calculate the log2 fold change and the adjusted *P* value of each gene between control and treatment.

### Phylogenetic trees of CODA, CYP76AD, cDOPA5GT, and betanidin 5GT/6GT

For CYP76AD, three CYP76AD-α proteins (GenBank accession: HQ656026, HQ656025, and HQ656024) from^105^ combined with the other 151 CYP76AD proteins (KR376350–KR376501) from ref. ^80^ were used as queries to collect homologs from cactus plants.

DODA-α proteins of *Beta vulgaris* (GenBank accession: HQ656027), *Portulaca grandiflora* (AJ580598), and *Mirabilis jalapa* (AB435372) combined with the DODA protein sequences (GenBank accession: KR376141–KR376346) previously studied in ref. ^80^ were used as queries to collect homologs from cactus plants.

cDOPA5GT and betanidin 5GT/6GT proteins were collected from ref. ^106^ and ref. ^107^, and used as query to collect homologs from cactus plants.

Homologs were filtered with domain e-value < 10^−6^ and coverage > = 0.3 (alignment length/HMM length). Filtered full-length protein sequences were aligned by MAFFT^108^, and then the phylogenies were built by FastTree^109^ and visualized with iTOL server^110^.

## Supplementary information

Supplemental Methods

Supplemental Tables

## Data Availability

The raw DNA sequencing reads and the assembled genome of *Hylocereus undatus* cultivar “David Bowie” have been submitted to NCBI. The BioProject ID is PRJNA664414, and the BioSample ID is SAMN16213845. The Whole Genome Shotgun accession number is JACYFF000000000.
