# Supplementary material for: The chromosome-level genome of dragon fruit reveals whole-genome duplication and chromosomal co-localization of betacyanin biosynthetic genes
Source: Hortic Res. 2021 Mar 10;8:63. doi: 10.1038/s41438-021-00501-6 (PMC7943767; doi:10.1038/s41438-021-00501-6)
Supplement: Supplementary file 1 — Supplemental Methods [file 41438_2021_501_MOESM1_ESM.docx]

**Supplementary Method**

**The chromosome-level genome of dragon fruit reveals whole genome duplication and chromosomal co-localization of betacyanin biosynthetic genes**

*Jinfang Zheng^1^, Lyndel W. Meinhardt^2^, Ricardo Goenaga^3^, Dapeng Zhang^2,*^, Yanbin Yin^1,*^*

*^1^Nebraska Food for Health Center, Department of Food Science and Technology, University of Nebraska, Lincoln, NE 68588, USA*

*^2^Sustainable Perennial Crops Lab, USDA-ARS, Beltsville, MD, USA*

*^3^Tropical Agriculture Research Station, USDA-ARS, Puerto Rico, USA*

* corresponding authors

Yanbin Yin

Tel: 1-402-472-4303

Email: [yyin@unl.edu](mailto:yyin@unl.edu)

Dapeng Zhang

Tel: 1-301-504-7477

Email: [dapeng.zhang@usda.gov](mailto:dapeng.zhang@usda.gov)

#### Plant material, 10x library prep and sequencing

Stem (cladode) samples of *H. undatus* cultivar “David Bowie” (**Figure 1A**) were collected from the USDA-ARS Tropical Agriculture Research Station in Mayaquez, Puerto Rico. The cultivar produces white-fleshed fruit with sweet, tangy flavor. High molecular Genomic DNA (gDNA) was extracted with a CTAB protocol and adjusted to a concentration of 1.0 ng/µl and 1.25 ng of template gDNA was loaded on a Chromium Genome Chip. Whole genome sequencing libraries were prepared using Chromium Genome Library & Gel Bead Kit v.2 (10X Genomics, cat. 120258), Chromium Genome Chip Kit v.2 (10X Genomics, cat. 120257), Chromium i7 Multiplex Kit (10X Genomics, cat. 120262) and Chromium controller according to manufacturer’s instructions with one modification. Briefly, gDNA was combined with Master Mix, a library of Genome Gel Beads, and partitioning oil to create Gel Bead-in-Emulsions (GEMs) on a Chromium Genome Chip. The GEMs were isothermally amplified with primers containing an Illumina Read 1 sequencing primer, a unique 16-bp 10x bar-code and a 6-bp random primer sequence, and bar-coded DNA fragments were recovered for Illumina library construction. The amount and fragment size of post-GEM DNA was quantified prior to using a Bioanalyzer 2100 with an Agilent High sensitivity DNA kit (Agilent, cat. 5067-4626). Prior to Illumina library construction, the GEM amplification product was sheared on an E220 Focused Ultrasonicator (Covaris, Woburn, MA) to approximately 350bp (55 seconds at peak power = 175, duty factor = 10, and cycle/burst = 200). Then, the sheared GEMs were converted to a sequencing library following the 10X standard operating procedure. The library was quantified by qPCR with a Kapa Library Quant kit (Kapa Biosystems-Roche) and sequenced on a NovaSeq6000 sequencer (Illumina, San Diego, CA) with paired-end 150 bp reads.

**Chicago library preparation and sequencing**

Two Chicago libraries were prepared as described previously (Putnam et al, 2016). Briefly, for each library, ~500ng of HMW gDNA was reconstituted into chromatin *in vitro* and fixed with formaldehyde. Fixed chromatin was digested with DpnII, the 5’ overhangs filled in with biotinylated nucleotides, and then free blunt ends were ligated. After ligation, crosslinks were reversed and the DNA purified from protein. Purified DNA was treated to remove biotin that was not internal to ligated fragments. The DNA was then sheared to ~350 bp mean fragment size and sequencing libraries were generated using NEBNext Ultra enzymes and Illumina-compatible adapters. Biotin-containing fragments were isolated using streptavidin beads before PCR enrichment of each library. The libraries were sequenced on an Illumina HiSeq X. The number and length of read pairs produced for each library was: 145 million, 2x150 bp for library 1; 181 million, 2x150 bp for library 2; Together, these Chicago library reads provided 235.75 x physical coverage of the genome (1-100 kb pairs).

**Dovetail HiC library preparation and sequencing**

Two Dovetail HiC libraries were prepared in a similar manner as described previously ^1^. Briefly, for each library, chromatin was fixed in place with formaldehyde in the nucleus and then extracted. Fixed chromatin was digested with DpnII, the 5’ overhangs filled in with biotinylated nucleotides, and then free blunt ends were ligated. After ligation, crosslinks were reversed and the DNA purified from protein. Purified DNA was treated to remove biotin that was not internal to ligated fragments. The DNA was then sheared to ~350 bp mean fragment size and sequencing libraries were generated using NEBNext Ultra enzymes and Illumina-compatible adapters. Biotin-containing fragments were isolated using streptavidin beads before PCR enrichment of each library. The libraries were sequenced on an Illumina HiSeq X. The number and length of read pairs produced for each library was: 204 million, 2x150 bp for library 1; 177 million, 2x150 bp for library 2. Together, these Dovetail HiC library reads provided 6,171.67 x physical coverage of the genome (10-10,000 kb pairs).

**Scaffolding the assembly with HiRise**

The input *de novo* assembly, shotgun reads, Chicago library reads, and Dovetail HiC library reads were used as input data for HiRise, a software pipeline designed specifically for using proximity ligation data to scaffold genome assemblies ^2^. An iterative analysis was conducted. First, Shotgun and Chicago library sequences were aligned to the draft input assembly using a modified SNAP read mapper (<http://snap.cs.berkeley.edu>). The separations of Chicago read pairs mapped within draft scaffolds were analyzed by HiRise to produce a likelihood model for genomic distance between read pairs, and the model was used to identify and break putative misjoins, to score prospective joins, and make joins above a threshold. After aligning and scaffolding Chicago data, Dovetail HiC library sequences were aligned and scaffolded following the same method. After scaffolding, shotgun sequences were used to close gaps between contigs.

#### Repeat and noncoding RNA annotation

RepeatModeler ^3^ and RepeatMasker ^4^ were employed to annotate repetitive elements in the draft dragon fruit genome and other cactus genomes. tRNA-scan2 ^5^ was used to identify tRNA genes. Infernal package ^6^ and Rfam ^7^ were employed to identify non-coding RNA genes. Specifically, Rfam contains noncoding RNA families represented by CM (covariance model) format. CMsearch scans input genome using these CMs for significant hits (e-value < 10^-6^).

#### Protein-coding gene prediction

MAKER ^8^ was employed to predict protein-coding genes by combining *ab initio* and homology-based approaches. Specifically, for homology-based gene finding, MAKER allows transcriptome and protein evidence as input. *H. polyrhizus* RNA-seq raw reads (SRR3234546) were downloaded from NCBI, and then trimmed with Trim_galore ^9^. The clean reads were assembled into transcriptome using Trinity ^10^ with the draft genome as the reference. Additionally, protein sequences of *A. thaliana* were downloaded from Phytozome ^11^, and protein sequences of cactus plants were downloaded from UniProt ^12^. Cactus proteins sequences were obtained by using “cactus” as keyword to search the UniProt database. For *ab initio* gene prediction, MAKER integrates results from SNAP ^13^ and Augustus ^14^. In this study MAKER was run three iterations to achieve a better accuracy. Trinity transcripts and proteins sequences of *A. thaliana* and UniProt were fed into MAKER during the first round. The result of first round were used to train Augustus models with the help of BUSCO ^15^, and to train SNAP models with the gene sequences extracted by maker2zff, a command of MAKER. During the second round, the Augustus model and SNAP models were fed into MAKER for *ab initio* gene prediction. After that, another round of Augustus and SNAP models were trained and run using the output of second round with the same methods described above. The result of this third round of MAKER run was used as the final protein-coding gene models.

In addition to *H. undatus*, MAKER gene predictions were also performed on four of the five cactus draft genomes sequenced by ^16^ except for *C. gigantea* following the same procedure as described above for *H. undatus*. No RNA-seq data was available for these four species. However, proteins sequences of *H. undatus* and *C. gigantea* (kindly provided by Dr. Sanderson MJ) were used as protein evidence for homology search. The redundant proteins from MAKER predictions were removed using seqkit ^17^, so were proteins < 50 aa.

#### Orthologous gene clusters and phylogenetic analyses

Proteins of a total of 16 sequenced plant genomes were selected to define orthologous gene clusters (OGCs) and for phylogenetic analyses. These genomes include the three C3 plants: *O. sativa ^18^, Cannabis sativa* ^19^*,* *A. thaliana ^20^*; three C4 plants: *Z. mays ^21^,* *S. bicolor ^22^, S. italica ^23^*; and ten CAM plants: *K. fedtschenkoi* ^24^*, S. album* ^25^*,* *C. gigantea, P. pringlei, L. schottii, S. thurberi,* *H. undatus,* *P. humboldtii**, A. comosus* ^26^*, P. equestris* ^27^. Six of the 10 CAM plants are cacti and their proteins were obtained by the processes described above. Proteins from *S. album* were provided by the author of ^25^. Proteins from all the other species were downloaded from Phyozome ^11^ or websites provided in their papers. Some genomes have proteins from alternative splicing, and such genomes were processed to only keep the longest isoform protein of each gene.

Proteins of the 16 genomes were combined as input to OrthoFinder ^28^, which generates orthologous gene clusters (OGCs) with the alignment tool MMseqs ^29^. All of the single-copy orthologs (OGCs containing a single copy of gene from each of the 16 genomes) were aligned with MUSCLE ^30^. The alignments of single-copy OGCs were concatenated into one super alignment, which was further processed by Gblocks ^31^. A phylogenetic tree was built using RAxML ^32^ to represent the species tree with 100 times of bootstrap and the evolutionary model -m PROTGAMMAJTT.

The divergence time of the 16 plants was estimated by r8s ^33^ with three calibrations: (i) the divergent time of *O. sativa* and *S. bicolor* = 50 MYA; (ii) the divergence time of *Cannabia* and *A.thaliana* = 106 MYA; and (iii) the divergence time of *A.comosus* and *P.equestris* = 114 MYA. These calibration times are obtained from the TimeTree database ^34^. The n-site is the length of the concatenated alignment of all single-copy OGCs. The input tree to r8s was the tree built by RAxML ^32^.

All the OGCs generated by OrthoFinder were analyzed by CAFE ^35^ to identify significantly expanded and contracted OGCs in different nodes of the species tree. The input time tree was built by r8s. All the species were assumed to evolved with one birth-death rate. OGCs with a large number of members (>= 100) were excluded from the analysis, as suggested by the CAFE tutorial.

#### GO and KEGG enrichment analysis

OGCs generated by OrthoFinder were classified into different groups according to what plant species that a CGC contains proteins from. For example, cactus-specific OGCs contain protein members from at least two cactus plants but not from non-cactus plants. For GO and KEGG annotation, all the proteins within a CGC were BlastP searched against the eggNOG database ^36^. The eggNOG hit contains GO term and KEGG term, which were transferred to the query protein. For each CGC, duplicated GO terms or KEGG terms were only counted once for the enrichment analysis. The Python package GOATOOLS ^37^ was used to convert all the GO terms to the 5th level. The procedure for statistical enrichment analysis was described in our recent paper ^38^. Briefly, for each GO or KEGG term, a binomial test P-value will be computed with foreground and background term counts as input. See the Result section for how we have defined foreground and background datasets depending on what questions we wanted to address. The “binom_test” function in the Python package scipy ^39^ was used for p-value calculation, and the function “p.adjust” in R language was used to adjust the p-value for multiple tests. An adjusted p-value (q-value) <= 0.01 indicates a significant enrichment in the foreground. The same approach had been used in our previous papers ^38,40^.

#### Whole genome duplication analysis

To examine the whole genome duplication (WGD) in cactus species, wgd ^41^ and MCScanX ^42^ were used to analyze the synteny and calculate synonymous substitution rates (Ks). Wgd is an integrated Python package using BlastP ^43^ to perform all-vs-all alignment of CDS sequences within a species. Then, MCL clustering is used to generate paralogous gene groups. After that, MAFFT ^44^ is used to align CDSs and protein sequences translated from these CDSs of a paralogous gene group. A phylogenetic tree is built on the alignment by FastTree ^45^. Finally, codeml in PAML package ^46^ is used to calculate Ks.

**RNA-seq data analysis**

The control (SRR8327214) and trypsin treated (SRR8327215) raw-reads were downloaded from NCBI SRA database and trimmed with Trim_galore ^9^. Then the clean reads were aligned to the dragon fruit draft genome by HISAT2 with default parameters ^47^. And then StringTie was used to assemble the transcripts following ^48^ and the read count was calculated with the Python script (prepDE.py) provide within Stringtie. Due to biological replicates were required for DEseq2 ^49^ analysis, we partitioned the raw reads of each sample into three subsamples as “biological” replicates. Then in total six subsamples were fed into DEseq2 to calculate the log2 fold change and adjusted p-value of each gene between control and treated subsamples. The up-regulated genes (p.adjusted < 0.05, log2FoldChange > 1) and down-regulated genes (p.adjusted < 0.05, log2FoldChange < -1) were filtered out to conduct the GO enrichment analysis as described in section “GO and KEGG enrichment analysis” for all the genes predicted by MAKER.

**Phylogenetic trees of CODA, CYP76AD, cDOPA5GT and betanidin 5GT/6GT**

For CYP76AD, three CYP76AD-α proteins (GenBank accession: HQ656026, HQ656025 and HQ656024) from ^50^ combined with the other 151 CYP76AD proteins (KR376350 - 376501) from ^51^ were used to build an HMM. Then the HMM was used to search against proteins of cactus plants including assembled UniGenes from RNA-seq of ^52^. The result was filtered with domain evalue < 10^-6^ and coverage >= 0.3 (alignment length/HMM length). Combined with the 151 CYP76AD proteins already analyzed in ^51^, the filtered full-length sequences were aligned by MAFFT, and then the phylogeny was built by FastTree.

DODA-α genes of *Beta vulgaris* (GenBank accession: HQ656027), *Portulaca grandiflora* (AJ580598) and *Mirabilis jalapa* (AB435372) combined with the DODA protein sequences (GenBank accession: KR376141 - KR376346) previously studied in ^51^ were used to build an HMM, which was used to search against the proteins of cactus plants including assembled UniGenes from RNA-seq of ^52^. The result was filtered with domain evalue < 10^-6^ and coverage >= 0.3 (alignment length/HMM length). Combined with the DODA protein sequences studied in ^51^, the phylogeny of DODA was built using MAFFT to generate multiple sequence alignment and FastTree to compute the tree.

cDOPA5GT and betanidin 5GT/6GT proteins were collected from ^53^ and ^54^, respectively. A total of 17 cDOPA5GT genes were used to build an HMM, and 13 betanidin 5GT/6GT proteins were used to build another HMM. Both HMMs were searched against cactus plants including assembled UniGenes from RNA-seq of ^52^. The results were filtered with domain evalue < 10^-6^ and coverage >= 0.3 (alignment length/HMM length). Considering the results from the two HMM searches shared many protein hits, homologs were combined to generate a single phylogeny.

1. Putnam, N.H. *et al.* Chromosome-scale shotgun assembly using an in vitro method for long-range linkage. *Genome Res* **26**, 342-50 <http://dx.doi.org/10.1101/gr.193474.115> (2016).

2. Lieberman-Aiden, E. *et al.* Comprehensive mapping of long-range interactions reveals folding principles of the human genome. *Science* **326**, 289-93 <http://dx.doi.org/10.1126/science.1181369> (2009).

3. Smit, A.F. & Hubley, R. RepeatModeler Open-1.0. (2010).

4. Smit, A., Hubley, R. & Green, P.J.D.D. RepeatMasker Open-4.0 (<http://www>. repeatmasker. org). (2018).

5. Chan, P.P. & Lowe, T.M. tRNAscan-SE: Searching for tRNA Genes in Genomic Sequences. *Methods Mol Biol* **1962**, 1-14 <http://dx.doi.org/10.1007/978-1-4939-9173-0_1> (2019).

6. Nawrocki, E.P. & Eddy, S.R. Infernal 1.1: 100-fold faster RNA homology searches. *Bioinformatics* **29**, 2933-5 <http://dx.doi.org/10.1093/bioinformatics/btt509> (2013).

7. Kalvari, I. *et al.* Rfam 13.0: shifting to a genome-centric resource for non-coding RNA families. *Nucleic Acids Res* **46**, D335-D342 <http://dx.doi.org/10.1093/nar/gkx1038> (2018).

8. Cantarel, B.L. *et al.* MAKER: an easy-to-use annotation pipeline designed for emerging model organism genomes. *Genome Res* **18**, 188-96 <http://dx.doi.org/10.1101/gr.6743907> (2008).

9. Krueger, F.J.A.w.t.a.C., quality, F.t.c.a. & files, a.t.t.F. Trim galore. **516**, 517 (2015).

10. Grabherr, M.G. *et al.* Full-length transcriptome assembly from RNA-Seq data without a reference genome. *Nat Biotechnol* **29**, 644-52 <http://dx.doi.org/10.1038/nbt.1883> (2011).

11. Goodstein, D.M. *et al.* Phytozome: a comparative platform for green plant genomics. *Nucleic Acids Res* **40**, D1178-86 <http://dx.doi.org/10.1093/nar/gkr944> (2012).

12. UniProt, C. UniProt: a worldwide hub of protein knowledge. *Nucleic Acids Res* **47**, D506-D515 <http://dx.doi.org/10.1093/nar/gky1049> (2019).

13. Korf, I. Gene finding in novel genomes. *BMC Bioinformatics* **5**, 59 <http://dx.doi.org/10.1186/1471-2105-5-59> (2004).

14. Stanke, M. *et al.* AUGUSTUS: ab initio prediction of alternative transcripts. *Nucleic Acids Res* **34**, W435-9 <http://dx.doi.org/10.1093/nar/gkl200> (2006).

15. Simao, F.A., Waterhouse, R.M., Ioannidis, P., Kriventseva, E.V. & Zdobnov, E.M. BUSCO: assessing genome assembly and annotation completeness with single-copy orthologs. *Bioinformatics* **31**, 3210-2 <http://dx.doi.org/10.1093/bioinformatics/btv351> (2015).

16. Copetti, D. *et al.* Extensive gene tree discordance and hemiplasy shaped the genomes of North American columnar cacti. *Proc Natl Acad Sci U S A* **114**, 12003-12008 <http://dx.doi.org/10.1073/pnas.1706367114> (2017).

17. Shen, W., Le, S., Li, Y. & Hu, F. SeqKit: A Cross-Platform and Ultrafast Toolkit for FASTA/Q File Manipulation. *PLoS One* **11**, e0163962 <http://dx.doi.org/10.1371/journal.pone.0163962> (2016).

18. Ouyang, S. *et al.* The TIGR Rice Genome Annotation Resource: improvements and new features. *Nucleic Acids Res* **35**, D883-7 <http://dx.doi.org/10.1093/nar/gkl976> (2007).

19. McKernan, K.J. *et al.* Sequence and annotation of 42 cannabis genomes reveals extensive copy number variation in cannabinoid synthesis and pathogen resistance genes. 2020.01.03.894428 <http://dx.doi.org/10.1101/2020.01.03.894428> %J bioRxiv (2020).

20. Lamesch, P. *et al.* The Arabidopsis Information Resource (TAIR): improved gene annotation and new tools. *Nucleic Acids Res* **40**, D1202-10 <http://dx.doi.org/10.1093/nar/gkr1090> (2012).

21. Hirsch, C.N. *et al.* Draft Assembly of Elite Inbred Line PH207 Provides Insights into Genomic and Transcriptome Diversity in Maize. *Plant Cell* **28**, 2700-2714 <http://dx.doi.org/10.1105/tpc.16.00353> (2016).

22. McCormick, R.F. *et al.* The Sorghum bicolor reference genome: improved assembly, gene annotations, a transcriptome atlas, and signatures of genome organization. *Plant J* **93**, 338-354 <http://dx.doi.org/10.1111/tpj.13781> (2018).

23. Bennetzen, J.L. *et al.* Reference genome sequence of the model plant Setaria. *Nat Biotechnol* **30**, 555-61 <http://dx.doi.org/10.1038/nbt.2196> (2012).

24. Yang, X. *et al.* The Kalanchoe genome provides insights into convergent evolution and building blocks of crassulacean acid metabolism. *Nat Commun* **8**, 1899 <http://dx.doi.org/10.1038/s41467-017-01491-7> (2017).

25. Wai, C.M. *et al.* Time of day and network reprogramming during drought induced CAM photosynthesis in Sedum album. *PLoS Genet* **15**, e1008209 <http://dx.doi.org/10.1371/journal.pgen.1008209> (2019).

26. Ming, R. *et al.* The pineapple genome and the evolution of CAM photosynthesis. *Nat Genet* **47**, 1435-42 <http://dx.doi.org/10.1038/ng.3435> (2015).

27. Cai, J. *et al.* The genome sequence of the orchid Phalaenopsis equestris. *Nat Genet* **47**, 65-72 <http://dx.doi.org/10.1038/ng.3149> (2015).

28. Emms, D.M. & Kelly, S. OrthoFinder: phylogenetic orthology inference for comparative genomics. *Genome Biol* **20**, 238 <http://dx.doi.org/10.1186/s13059-019-1832-y> (2019).

29. Steinegger, M. & Soding, J. MMseqs2 enables sensitive protein sequence searching for the analysis of massive data sets. *Nat Biotechnol* **35**, 1026-1028 <http://dx.doi.org/10.1038/nbt.3988> (2017).

30. Edgar, R.C. MUSCLE: multiple sequence alignment with high accuracy and high throughput. *Nucleic Acids Res* **32**, 1792-7 <http://dx.doi.org/10.1093/nar/gkh340> (2004).

31. Castresana, J. Selection of conserved blocks from multiple alignments for their use in phylogenetic analysis. *Mol Biol Evol* **17**, 540-52 <http://dx.doi.org/10.1093/oxfordjournals.molbev.a026334> (2000).

32. Stamatakis, A. RAxML version 8: a tool for phylogenetic analysis and post-analysis of large phylogenies. *Bioinformatics* **30**, 1312-3 <http://dx.doi.org/10.1093/bioinformatics/btu033> (2014).

33. Sanderson, M.J. r8s: inferring absolute rates of molecular evolution and divergence times in the absence of a molecular clock. *Bioinformatics* **19**, 301-2 <http://dx.doi.org/10.1093/bioinformatics/19.2.301> (2003).

34. Kumar, S., Stecher, G., Suleski, M. & Hedges, S.B. TimeTree: A Resource for Timelines, Timetrees, and Divergence Times. *Mol Biol Evol* **34**, 1812-1819 <http://dx.doi.org/10.1093/molbev/msx116> (2017).

35. De Bie, T., Cristianini, N., Demuth, J.P. & Hahn, M.W. CAFE: a computational tool for the study of gene family evolution. *Bioinformatics* **22**, 1269-71 <http://dx.doi.org/10.1093/bioinformatics/btl097> (2006).

36. Huerta-Cepas, J. *et al.* eggNOG 5.0: a hierarchical, functionally and phylogenetically annotated orthology resource based on 5090 organisms and 2502 viruses. *Nucleic Acids Res* **47**, D309-D314 <http://dx.doi.org/10.1093/nar/gky1085> (2019).

37. Klopfenstein, D.V. *et al.* GOATOOLS: A Python library for Gene Ontology analyses. *Sci Rep* **8**, 10872 <http://dx.doi.org/10.1038/s41598-018-28948-z> (2018).

38. Fitzek, E. *et al.* Cell Wall Enzymes in Zygnema circumcarinatum UTEX 1559 Respond to Osmotic Stress in a Plant-Like Fashion. *Front Plant Sci* **10**, 732 <http://dx.doi.org/10.3389/fpls.2019.00732> (2019).

39. Virtanen, P. *et al.* SciPy 1.0: fundamental algorithms for scientific computing in Python. *Nat Methods* <http://dx.doi.org/10.1038/s41592-019-0686-2> (2020).

40. Orton, L.M. *et al.* Zygnema circumcarinatum UTEX 1559 chloroplast and mitochondrial genomes provide insight into land plant evolution. *J Exp Bot* **71**, 3361-3373 <http://dx.doi.org/10.1093/jxb/eraa149> (2020).

41. Zwaenepoel, A. & Van de Peer, Y. wgd-simple command line tools for the analysis of ancient whole-genome duplications. *Bioinformatics* **35**, 2153-2155 <http://dx.doi.org/10.1093/bioinformatics/bty915> (2019).

42. Wang, Y. *et al.* MCScanX: a toolkit for detection and evolutionary analysis of gene synteny and collinearity. *Nucleic Acids Res* **40**, e49 <http://dx.doi.org/10.1093/nar/gkr1293> (2012).

43. Altschul, S.F., Gish, W., Miller, W., Myers, E.W. & Lipman, D.J. Basic local alignment search tool. *J Mol Biol* **215**, 403-10 <http://dx.doi.org/10.1016/S0022-2836(05)80360-2> (1990).

44. Katoh, K., Asimenos, G. & Toh, H. Multiple alignment of DNA sequences with MAFFT. *Methods Mol Biol* **537**, 39-64 <http://dx.doi.org/10.1007/978-1-59745-251-9_3> (2009).

45. Price, M.N., Dehal, P.S. & Arkin, A.P. FastTree 2--approximately maximum-likelihood trees for large alignments. *PLoS One* **5**, e9490 <http://dx.doi.org/10.1371/journal.pone.0009490> (2010).

46. Yang, Z. PAML: a program package for phylogenetic analysis by maximum likelihood. *Comput Appl Biosci* **13**, 555-6 <http://dx.doi.org/10.1093/bioinformatics/13.5.555> (1997).

47. Kim, D., Langmead, B. & Salzberg, S.L. HISAT: a fast spliced aligner with low memory requirements. *Nat Methods* **12**, 357-60 <http://dx.doi.org/10.1038/nmeth.3317> (2015).

48. Pertea, M., Kim, D., Pertea, G.M., Leek, J.T. & Salzberg, S.L. Transcript-level expression analysis of RNA-seq experiments with HISAT, StringTie and Ballgown. *Nat Protoc* **11**, 1650-67 <http://dx.doi.org/10.1038/nprot.2016.095> (2016).

49. Love, M.I., Huber, W. & Anders, S. Moderated estimation of fold change and dispersion for RNA-seq data with DESeq2. *Genome Biol* **15**, 550 <http://dx.doi.org/10.1186/s13059-014-0550-8> (2014).

50. Hatlestad, G.J. *et al.* The beet R locus encodes a new cytochrome P450 required for red betalain production. *Nat Genet* **44**, 816-20 <http://dx.doi.org/10.1038/ng.2297> (2012).

51. Brockington, S.F. *et al.* Lineage-specific gene radiations underlie the evolution of novel betalain pigmentation in Caryophyllales. *New Phytol* **207**, 1170-80 <http://dx.doi.org/10.1111/nph.13441> (2015).

52. Xiong, R. *et al.* Transcriptomic analysis of flower induction for long-day pitaya by supplementary lighting in short-day winter season. *BMC Genomics* **21**, 329 <http://dx.doi.org/10.1186/s12864-020-6726-6> (2020).

53. Sasaki, N. *et al.* Isolation and characterization of cDNAs encoding an enzyme with glucosyltransferase activity for cyclo-DOPA from four o'clocks and feather cockscombs. *Plant Cell Physiol* **46**, 666-70 <http://dx.doi.org/10.1093/pcp/pci064> (2005).

54. Vogt, T. Substrate specificity and sequence analysis define a polyphyletic origin of betanidin 5- and 6-O-glucosyltransferase from Dorotheanthus bellidiformis. *Planta* **214**, 492-5 <http://dx.doi.org/10.1007/s00425-001-0685-1> (2002).
